# Healthcare providers’ readiness to screen for intimate partner violence in obstetrics and gynecology units in Amhara regional state referral hospitals, Ethiopia: validation and cross-sectional survey using the DVHCPSS tool

**DOI:** 10.3389/fgwh.2025.1408703

**Published:** 2025-04-24

**Authors:** Kidist Ayalew Abebe, Tirusew Nigussie Kebede, Birhan Tsegaw Taye, Mulualem Silesh, Mesfin Tadese, Moges Sisay Chekol, Tesfanesh Lemma Demisse, Betel Bogale Workineh, Abayneh Akililu Solomon, Bayew Kelkay Rade, Getie Lake Aynalem

**Affiliations:** ^1^ Department of Midwifery, School of Nursing and Midwifery, College of Medicine and Health Sciences, Debre Birhan University, Debre Birhan, Ethiopia; ^2^ Department of Midwifery, School of Nursing and Midwifery, Institute of Health Sciences, Wollega University, Nekemte, Ethiopia; ^3^ Department of Clinical Midwifery, School of Midwifery, College of Medicine and Health Sciences, University of Gondar, Gondar, Ethiopia

**Keywords:** readiness, intimate partner violence, healthcare provider, referral hospitals, Ethiopia

## Abstract

**Introduction:**

Intimate partner violence (IPV) is most prevalent among women of reproductive age and can have lifelong consequences. Screening within healthcare settings represents a promising first step toward addressing IPV, with healthcare providers playing a central role in this response. A lack of healthcare provider readiness to screen for IPV may leave victims vulnerable to continued physical, psychological, sexual, and reproductive health problems. This study aimed to assess the readiness of healthcare providers to screen for IPV and to identify factors affecting screening practices in obstetrics and gynecology units of referral hospitals in Amhara regional state, Ethiopia.

**Methods:**

An institution-based cross-sectional study was conducted between 9 January and 4 February 2023. A simple random sampling technique was employed to select study participants. A pilot study was conducted to assess the reliability and construct validity of the tool, and data were collected using a self-administered questionnaire. The data were entered into EPI-Data version 4.6 and analyzed using STATA version 14. Bivariable and multivariable logistic regression models were applied to identify associated factors.

**Result:**

From 409 study participants, 46.5% [95% confidence interval (CI): 42–51] were ready to screen for IPV among reproductive-aged women. Being male [adjusted odds ratio (AOR) = 1.64, 95% CI: 1.03–2.61], trained in IPV (AOR = 2.84, 95% CI: 1.64–4.94), favorable attitude toward IPV screening (AOR = 2.21, 95% CI: 1.42–3.44), good knowledge of IPV (AOR = 2.23, 95% CI: 1.42–3.50), and availability of IPV guidelines in their working area (AOR = 1.74, 95% CI: 1.07–2.81) were found to be significantly associated factors with healthcare providers’ readiness to screen for IPV.

**Conclusion:**

In this study, fewer than half of the healthcare providers were found to be ready to screen for IPV. Factors that significantly influenced their readiness included the availability of training on IPV, positive attitudes toward IPV screening, adequate knowledge about IPV, and access to IPV screening guidelines within their work environment.

## Introduction

Intimate partner violence (IPV) is a preventable public health issue (1) that encompasses physical violence, sexual violence (SV), stalking, and psychological harm inflicted by a current or former intimate partner (2). It affects millions of women globally, regardless of age, economic status, race, religion, ethnicity, or educational background. Although IPV can affect women of all ages, it is more common among those of reproductive age and is associated with gynecological disorders and pregnancy complications (1). Globally, one in three women experiences physical and/or sexual violence by an intimate partner, or sexual violence by a non-partner, at some point in her life (3).

The World Health Organization (WHO) multi-country study on women’s health and domestic violence undertaken in developing settings, including Ethiopia, confirmed that physical and sexual partner violence against women is widespread, with prevalence in the range of 15%–71% (4). In sub-Saharan Africa, the lifetime prevalence of IPV is 37%, making it one of the world’s most seriously impacted regions (5). In Ethiopia, more than one-third of ever-married women had been subjected to IPV by their husbands or partners at some point in their lives, and in the Amhara region, IPV is prevalent in 35% of women (6).

Although IPV rates vary across low-, middle-, and high-income regions, its health consequences are similar worldwide (7). IPV has lifelong repercussions, including emotional trauma, lasting physical impairment, chronic health conditions, and even death. It is also linked to sexually transmitted infections, unplanned pregnancies, and unsafe abortions, which are serious public health consequences of IPV (1). During pregnancy, IPV can lead to miscarriage, premature labor and delivery, low birth weight, maternal depression, delaying prenatal care, insufficient weight gain during pregnancy, substance abuse, and reduced breastfeeding rates (8). Furthermore, children born to mothers who experience IPV face higher risks of poor growth and development, contributing to increased under-5 mortality rates (9).

The 2030 Sustainable Development Agenda identifies the elimination of all forms of violence against women and girls in the public and private spheres as a key target (10). In addition, the Ethiopian Strategic Plan from 2021 to 2025 stated that improving health workers’ competency in the prevention of and response to gender-based violence/SV (GBV/SV) is a crucial output in making the health system more responsive (11).

According to the American College of Obstetricians and Gynecologists (ACOG), all patients should be screened during annual examinations, family planning, and preconception visits. Screening for pregnant women should take place at various times throughout the pregnancy, including the initial prenatal visit, at least once per trimester, and at the postpartum checkup (1). In addition, the United States Preventive Services Task Force (USPSTF) and the American Nurses Association (ANA) also recommend screening all women of childbearing age for IPV and providing services for those who screen positive (12). However, studies indicated that IPV screening is not always done by healthcare providers (HCPs), often because of insufficient preparedness (13).

A HCP is likely to be the first professional contact for victims of IPV, especially obstetrics and gynecology (OBY/GYN) healthcare providers, who serve a vital role in women’s healthcare and have a unique opportunity to identify and support women experiencing IPV. This is because the nature of the patient–provider relationship holds ample opportunity for interventions (14, 15). Women often attend multiple visits during preconception, pregnancy, and postpartum care. These visits typically involve routine discussions about a wide range of health issues, including reproductive, sexual, and mental health, creating an ongoing opportunity for providers to identify IPV and offer help in a safe and confidential environment (15). Moreover, incorporating IPV screening and intervention into OBY/GYN practice aligns with global and national health priorities, including the Sustainable Development Goals (SDGs) and Ethiopia’s Health Sector Transformation Plan, both of which prioritize maternal and child health. By addressing IPV within these units, HCPs not only support the immediate safety and wellbeing of mothers and infants but also contribute to long-term improvements in family health and stability (16, 17).

**Figure 1:**
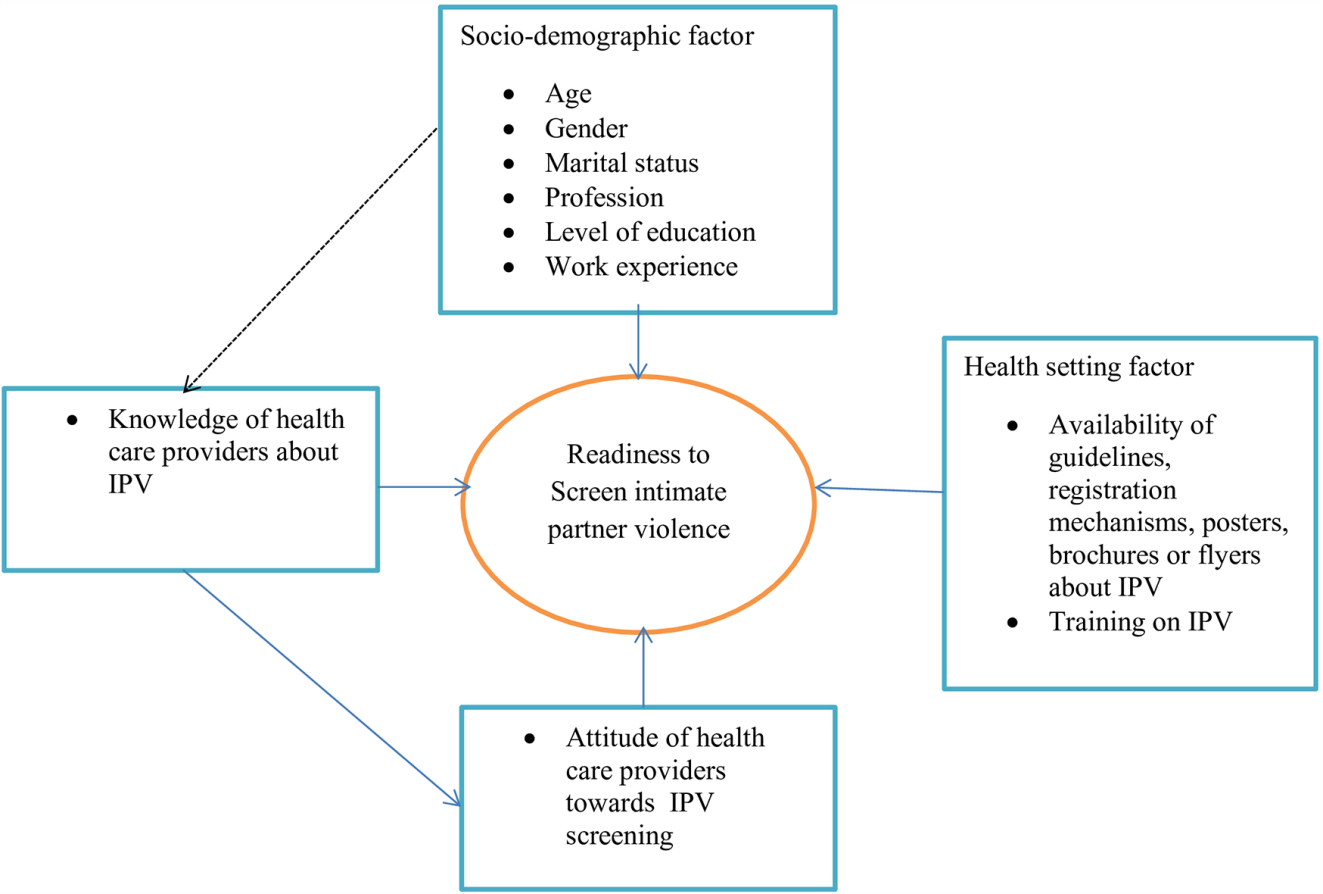
Conceptual framework adapted from studies (22–25) for factors affecting healthcare providers’ readiness to screen for intimate partner violence among reproductive-aged women in obstetrics and gynecology units of Amhara regional state referral hospitals, 2023.

Screening is a promising first step toward addressing the issue of IPV in healthcare settings (18). A lack of healthcare provider readiness to screen for IPV may leave victims vulnerable to continued physical, psychological, sexual, and reproductive health issues (19). Despite the fact that adequate knowledge, attitudes, and skills regarding injury treatment, referral systems, and legal rights information are critical for the early detection and intervention of IPV (20), the majority of HCPs do not see IPV assessment and management as part of their role, and most believe they lack knowledge about what questions to ask or how to respond if a woman reports being abused, as well as skills in the area of IPV (21). Different evidence showed that HCPs’ sociodemographic characteristics, knowledge of IPV, attitudes toward IPV screening, and healthcare facility setting affect their readiness to screen for IPV (Figure 1) (22–25).

Despite being a widespread and critical public health issue in Ethiopia – with studies indicating that the study area has one of the highest IPV prevalence rates (8, 26–28) – there is, to the best of the investigators’ knowledge, no evidence on IPV screening practices among HCPs in the country. Limited findings suggest that HCPs have minimal understanding of IPV’s severe effects on pregnancy outcomes and long-term health implications. Many HCPs face challenges in identifying IPV due to inadequate preparation, limited skills, and lack of experience, leading to hesitation in addressing signs of abuse, underreporting, and missed opportunities for early intervention (29–31). To implement effective and tailored interventions, it is crucial to first determine the readiness of HCPs and identify the factors influencing their ability to screen for IPV. However, to date, no studies have been conducted in the study area to address this gap. Therefore, this study aims to assess the readiness of HCPs and the associated factors influencing IPV screening in referral hospitals within Amhara regional state.

## Methods

### Study design, setting, and period

An institution-based cross-sectional study was conducted between 9 January and 4 February 2023. The study was conducted in referral hospitals in Amhara regional state, one of 11 national states in Ethiopia. Amhara has eight referral hospitals, namely University of Gondar Comprehensive Specialized Hospital (UOGCSH), Felege Hiwot Referral Hospital (FHRH), Tibebe Gion Comprehensive Specialized Hospital (TGCSH), Dessie Referral Hospital (DRH), Debre-Markos Comprehensive Specialized Hospital (DMCSH), Debre Birhan Referral Hospital (DBRH), Debre Tabor Comprehensive Specialized Hospital (DTCSH), and Woldiya Referral Hospital (WRH). Each referral hospital’s catchment population is estimated to be 5–7 million people (32).

### Study population and eligibility criteria

#### Source population

All healthcare providers working in the OBY/GYN unit in Amhara regional state referral hospitals were included in the study.

#### Study population

All healthcare providers working in the OBY/GYN unit in Amhara regional state referral hospitals were available during the data collection period.

#### Inclusion criteria

The inclusion criteria included healthcare providers, working in the OBY/GYN unit in Amhara regional state referral hospitals for at least 6 months.

#### Exclusion criteria

The exclusion criteria were healthcare providers from the OBY/GYN unit who were on maternity leave, on long- or short-term training, and on sick leave during the data collection period.

#### Sample size determination

The sample size was determined using the single population proportion formula by considering the following assumptions: the proportion of healthcare providers who are ready to screen for intimate partner violence is 50% (since there was no previous study), a 95% confidence level, and a 5% margin of error:

$$n=\frac{{\left(Z\alpha /2\right)}^{2}\;*\;p(1-p)}{d^{2}}$$

where *n* is the desirable sample size, *Z* is the standard normal distribution curve value for the 95% confidence level = 1.96, *P* is the proportion of readiness to screen for intimate partner violence against reproductive-aged women among healthcare providers, and *d* is the margin of error. Assuming a 10% non-response rate, the minimum adequate sample size was 423.

#### Sampling procedure

All eight referral hospitals in Amhara regional state were included. The sample size was proportionally allocated for each referral hospital. Lastly, healthcare providers working in the OBY/GYN unit were selected by a simple random sampling technique using the lottery method with their lists as a sampling frame.

### Study variables

#### Outcome variable

The outcome variable was the readiness of healthcare providers’ working in the OBY/GYN unit to screen for IPV.

#### Independent variables

Independent variables were as follows: sociodemographic factors, including age, gender, marital status, religion, profession, level of education, work experience; knowledge of healthcare providers about IPV; attitude of healthcare providers toward IPV screening; and health setting factors (availability of IPV guidelines, registration mechanisms for IPV cases, training on IPV).

### Operational definition

#### Intimate partner violence

IPV is defined as physical and/or sexual and/or psychological violence committed to women by boyfriends, cohabitants, and husbands (2).

#### Readiness to screen for IPV

Readiness to screen for IPV was measured using the Domestic Violence Healthcare Provider Survey Scale (DVHCPSS) instrument, which has the following domains: “perceived self-efficacy,” “professional role resistance/fear of offending the patient,” “blame victim,” “system support,” and “victim/provider safety” (33). HCP scores at or above the mean of the composite items were categorized as “ready” to screen for IPV.

“Perceived self-efficacy” (six items) assesses HCPs’ own perceived efficacy in inquiring about IPV, and the higher the individual score, the higher the perceived self-efficacy (33). A higher score indicated a more favorable outcome (34); therefore, a score at or above the mean signified that the HCP had good perceived self-efficacy.

“System support” (four items) assesses HCPs’ access to and confidence in the availability of social and psychiatric support services. A higher individual score indicates greater perceived system support (33); therefore, a higher score indicated a more favorable outcome (34); therefore, a score at or above the mean signified that the HCP had adequate access to system support services.

“Professional role resistance/fear of offending clients” (six items) assesses HCPs’ opinions on whether inquiries about IPV may conflict with ethical issues governing their communication with clients. A higher individual score indicates greater resistance or fear of offending the patient (33). A lower score indicated a more favorable outcome (34); therefore, a score below the mean signified that the HCP had a low professional role resistance/fear of offending the patient.

“Victim blame” (five items) assesses HCPs’ attitudes toward victims, and the higher the individual score, the higher the propensity to blame the victim (33). A lower score indicated a more favorable outcome (34); therefore, a score below the mean indicates that HCPs do not blame the victim for being abused.

“Victim/provider safety” (seven items) assesses HCPs’ perception on whether inquiring about IPV from batterers would further jeopardize victim/care provider safety, and the higher the individual score, the lower the concerns about victim/provider safety (33). A lower score indicated a more favorable outcome (34); therefore, a score below the mean indicates that HCPs had concerns for their own and their client’s safety.

#### Good knowledge about IPV

Healthcare providers were considered to have good knowledge about IPV if their scores on the knowledge-related questions were at or above the mean.

#### Favorable attitude toward IPV screening

HCPs were considered to have favorable attitudes toward IPV screening if their scores on the attitude-related questions were at or above the mean.

### Data collection tools and procedures

A pilot-tested, self-administered questionnaire adapted from various studies (22–25, 35) was used to collect data. It included questions on sociodemographic characteristics, DVHCPSS domains, knowledge-related variables, attitude-related variables, and health sector-related variables.

The DVHCPSS tool, which has been previously validated in different developing countries, was used to assess readiness to screen for IPV (35, 36), with the authors of these previous studies concluding that the tool could be used to assess HCPs’ readiness to screen for IPV. They also recommended that other researchers contextualize the tool to local circumstances. Studies were also conducted in Egypt using the DVHCPS tool (7). The DVHCPSS tool is categorized into five domains, with a total of 28 items through a 5-point Likert scale ranging from 1 (“strongly disagree”) to 5 (“strongly agree”). Among the five domains used to measure HCPs’ readiness to screen for IPV, lower scores were preferable for the blame victim domain items, professional role resistance/fear of offending the patient domain items, and victim/provider safety domain items, while higher score were preferable for the perceived self-efficacy and system support domain items (34). Therefore, based on summative scores designed to assess the prevalence of HCPs’ readiness to screen for IPV, the three domains in which lower scores were preferable were reversely recoded.

Furthermore, negatively phrased questions to assess the attitude and knowledge of HCPs were also reversely recoded.

The data were collected by eight BSc midwives, with the data collection process carefully supervised by three MSc midwives. One-day training was provided for the data collectors and supervisors to ensure they understood the study’s purpose, procedures, and data collection techniques. The session began with an explanation of the study’s aim and its significance in assessing healthcare providers’ readiness to screen for IPV, emphasizing the importance of their role in this process. The study procedures were then outlined, ensuring they understood the ethical considerations such as informed consent and confidentiality. The data collectors were trained on how to administer the DVHCPSS tool and how to check with participants as soon as possible to complete any missing and unclear responses, with a focus on consistency and accuracy in assessing healthcare providers’ readiness. The supervisors also provided training on how to closely monitor the data collection process and its completeness. To reinforce their learning, we conducted role-playing exercises where they practiced using the tool in mock scenarios, addressing any challenges they might face. The training concluded with a question and answer session to clarify any doubts, followed by an evaluation to ensure they were fully prepared to conduct data collection effectively and ethically.

### Data quality control

The questionnaire and consent documents were translated from English to Amharic and then retranslated back to English to ensure its consistency. A pretest was carried out on 5% of the sample size at Motta General Hospital to check the response, language clarity, and comprehension of the items. A pilot study was then conducted in Injibara and Finote Selam General Hospitals to check the tool’s construct validity and reliability, since the DVHCPSS tool for measuring healthcare providers’ readiness to screen for IPV was not validated among Ethiopian HCPs. Even if the tool was validated in other countries, it may not be reliable and valid in Ethiopia due to sociocultural and socioeconomic differences. To ensure high data quality during data collection, each data collector and supervisor received comprehensive training on the study objectives, data collection tool, and procedures. The supervisor conducted daily checks on completed questionnaires to verify completeness and address any missing or unclear responses promptly.

### Reliability and validity of the tool

Some studies conduct a pilot study on 10%–12% of the sample size (7, 37, 38). In this study, an external pilot was conducted among 10% (43 HCPs) of the calculated sample size. The pilot was carried out at Injibara and Finote Selam General Hospitals, selected for their accessibility, feasibility, and practicality in testing the tool’s validity and reliability. Although these facilities are not referral hospitals, they offer a wide range of healthcare services and share structural similarities with the main study sites, making them suitable for evaluating the DVHCPSS tool’s validity and reliability in a real-world setting.

By proportionally allocating the formula, 24 participants were from Injibara General Hospital and 19 participants from Finote Selam General Hospital. A total of 42 healthcare providers (response rate of 97.7%) working in the OBY/GYN unit participated in the pilot study.

In the confirmatory factor analysis conducted during the pilot study, the construct validity and reliability of the tool were checked.

The convergent and discriminatory validity of DVHCPSS was examined. Its convergent validity was checked to assess the level of correlation of the observed item with other measures of the same construct, while its discriminant validity was assessed to ensure that the constructs actually differ from one another to indicate they are not measuring the same thing (39).

Items with a factor loading of at least 0.30 and significant factors were considered (36). Then convergent validity was then examined through the average variance extracted (AVE). An AVE equal to or greater than 0.50 was used to determine the convergent validity (40).

The majority of the items in the original DVHCPSS exhibited significant factor loading except for the following items from different domains: one item from the perceived self-efficacy domain (I don’t have the time to ask about IPV in my practice), one item from the professional role resistance/fear of offending the patient domain (It is not my place to interfere with how a couple chooses to resolve conflicts), two items from the blame victim domain (People are only victims if they choose to be) and (When it comes to IPV victimization, it usually “two to tango”), and three items from the victim/provider safety domain (I feel I can discuss issues of battering and abuse with a battering patient without further endangering the victim), (I feel I can effectively discuss issues of battering and abuse with a battering patient), and (I feel there are ways of asking about battering behavior without placing myself at risk).

As a result, 7 items were removed from the total of 35, while 28 items have a value of more than 0.3 and significant factors loading in their corresponding domains.

The results for the convergent validity of the domains are as follows: perceived self-efficacy (AVE = 0.51), professional role resistance (AVE = 0.51), blaming the victim (AVE = 0.5), system support (AVE = 0.5), and victim/provider safety (AVE = 0.5). These results show that all domains are convergent validated (Table 1).

**Table 1:** Internal reliability, alpha if item deleted, and item-factor loading for each subscale of the DVHCPSS tool used to measure healthcare providers’ readiness to screen for IPV among reproductive-aged women in Amhara regional state referral hospitals, 2023.

Item	Item description	Item-factor loading	Alpha if the item deleted
Perceived self-efficacy domain			
1	There are strategies I can use to encourage batterers to seek help.	0.80	0.82
2	There are strategies I can use to help victims of IPV change their situation.	0.62	0.84
3	I feel confident that I can make appropriate referrals for batterers.	0.84	0.82
4	I feel confident that I can make the appropriate referrals for abused patients.	0.73	0.84
5	I have ready access to information detailing management of IPV.	0.53	0.85
6	There’re ways I can ask batterers about their behavior that will minimize risk to the potential victim	0.70	0.83
	Overall Cronbach’s alpha		**0**.**86**
	AVE	**0**.**51**	
Professional role resistance/fear of offending the patients domain			
7	Asking patients about IPV is an invasion of their privacy.	0.88	0.79
8	It is demeaning to patients to question them about abuse.	0.86	0.79
9	If I ask non-abused patients about IPV, they will get very angry.	0.58	0.84
10	I am afraid of offending the patient if I ask about IPV.	0.59	0.83
11	I think that investigating the underlying cause of a patient’s injury is not part of medical care.	0.68	0.83
12	If patients do not reveal abuse to me, then it is none of my business.	0.62	0.82
	Overall Cronbach’s alpha		**0**.**84**
	AVE	**0**.**51**	
Blame victim domain			
13	A victim must be getting something out of the abusive relationship, or else she would leave.	0.77	0.74
14	I have patients whose personalities cause them to be abused.	0.67	0.74
15	Women who choose to step out of traditional roles are a major cause of IPV.	0.69	0.75
16	The victim’s passive-dependent personality often leads to abuse.	0.68	0.76
17	The victim has often done something to bring about violence in the relationship.	0.65	0.75
	Overall Cronbach’s alpha		**0**.**79**
	AVE	**0**.**5**	
System support domain			
18	I have ready access to social workers or community advocates to assist in the management of IPV.	0.59	0.77
19	I feel that social work personnel can help manage IPV patients.	0.78	0.69
20	I have ready access to mental health services should our patients need referrals.	0.69	0.73
21	I feel that the mental health services at my clinic or agency can meet the needs to IPV victims.	0.74	0.73
	Overall Cronbach’s alpha		**0**.**78**
	AVE	**0**.**50**	
Victim/provider safety domain			
22	There is no way to ask batterers about their behaviors without putting the victims in more danger.	0.62	0.84
23	I am afraid if I talk to the batterer, I will increase risk for the victim.	0.84	0.81
24	I feel it is best to avoid dealing with the batterer out of fear and concern for the victim’s safety.	0.72	0.83
25	I am reluctant to ask batterers about their abusive behavior out of Concern for my personal safety.	0.65	0.83
26	There is not enough security at my work place to safely permit discussion of IPV with batterers.	0.55	0.84
27	I am afraid of offending patients if I ask about their abusive behavior.	0.72	0.82
28	When challenged, batterers frequently direct their anger toward healthcare providers.	0.62	0.84
	Overall Cronbach’s alpha		**0**.**85**
	AVE	**0**.**5**	

To determine the discriminatory validity, the square root of each construct’s AVE should exceed its correlation with other constructs (39). In this study, discriminant validity was confirmed, as all inter-factor correlations were lower than the square root of AVE for each corresponding construct (Table 2).

**Table 2:** Spearman rank correlation between DVHCPSS tool domains used to measure healthcare providers’ readiness to screen for IPV among reproductive-aged women in Amhara regional state referral hospitals, 2023.

Factors	Perceived self-efficacy	Professional role resistance/fear of offending the patients	Blame victim	System support	Victim/provider safety
Perceived self-efficacy	1.000				
Professional role resistance/fear of offending the patients	−0.108	1.000			
Blame victim	0.090	0.192	1.000		
System support	0.196	−0.144	0.006	1.000	
Victim/provider safety	0.186	0.315	0.245	0.279	1.000

The internal reliability of the DVHCPSS tool was measured using Cronbach’s alpha coefficient for each domain. This coefficient measures reliability based on the interrelationship among observed item variables designed to measure a single construct. A Cronbach’s alpha value greater than 0.7 was considered significant, indicating adequate internal reliability (41).

Cronbach’s alpha coefficient for the overall DVHCPSS tool was 0.81. Each subscale (domain) demonstrated acceptable internal reliability in the range of 0.78–0.86. The alpha values for the domain items were in the range of 0.82–0.85 for perceived self-efficacy, 0.79–0.84 for professional role resistance, 0.74–0.76 for blaming the victim, 0.69–0.77 for system support, and 0.81–0.84 for victim/provider safety (Table 1).

### Data processing and analysis

The collected data were manually checked for completeness, and incomplete data were excluded from the analysis. Then, the data were coded, recoded, and entered into EPI-Data version 4.6 and exported to STATA 14 for analysis. Frequencies, proportions, and summaries of descriptive statistics were employed to describe the study population in relation to relevant variables.

A binary logistic regression model was fitted to identify independent variables associated with the outcome. Variables with a *p*-value of ≤0.25 in the bivariable analysis were proceeded to the multivariable logistic regression to handle the effect of possible confounders. The Hosmer–Lemeshow test was used to assess how well the logistic regression model fit the observed data, producing a non-significant *p*-value (*p* > 0.05), indicating that the model provided an adequate fit to the data. In multivariable analysis, a *p*-value of ≤0.05 with a 95% confidence interval (CI) for odds ratio was used to determine significant association.

### Ethical considerations

Ethical clearance was obtained from the University of Gondar’s School of Midwifery’s ethical review committee (reference number MIDW/30/2015). A written permission letter was also received from hospital managers and ward coordinators in the study settings. Before data collection began, the study participants were informed about the objective and purpose of the study. They were assured that their participation was completely voluntary and that all information would be kept strictly confidential. Participants were approached individually and provided with detailed information about the study. Written informed consent was then obtained from each study participant. No incentives were provided for participation. To preserve confidentiality, the data were not exposed to any third party except the investigators. All necessary methods were carried out in accordance with ethical guidelines and regulations.

## Results

### Sociodemographic characteristics of study participants

**Figure 2:**
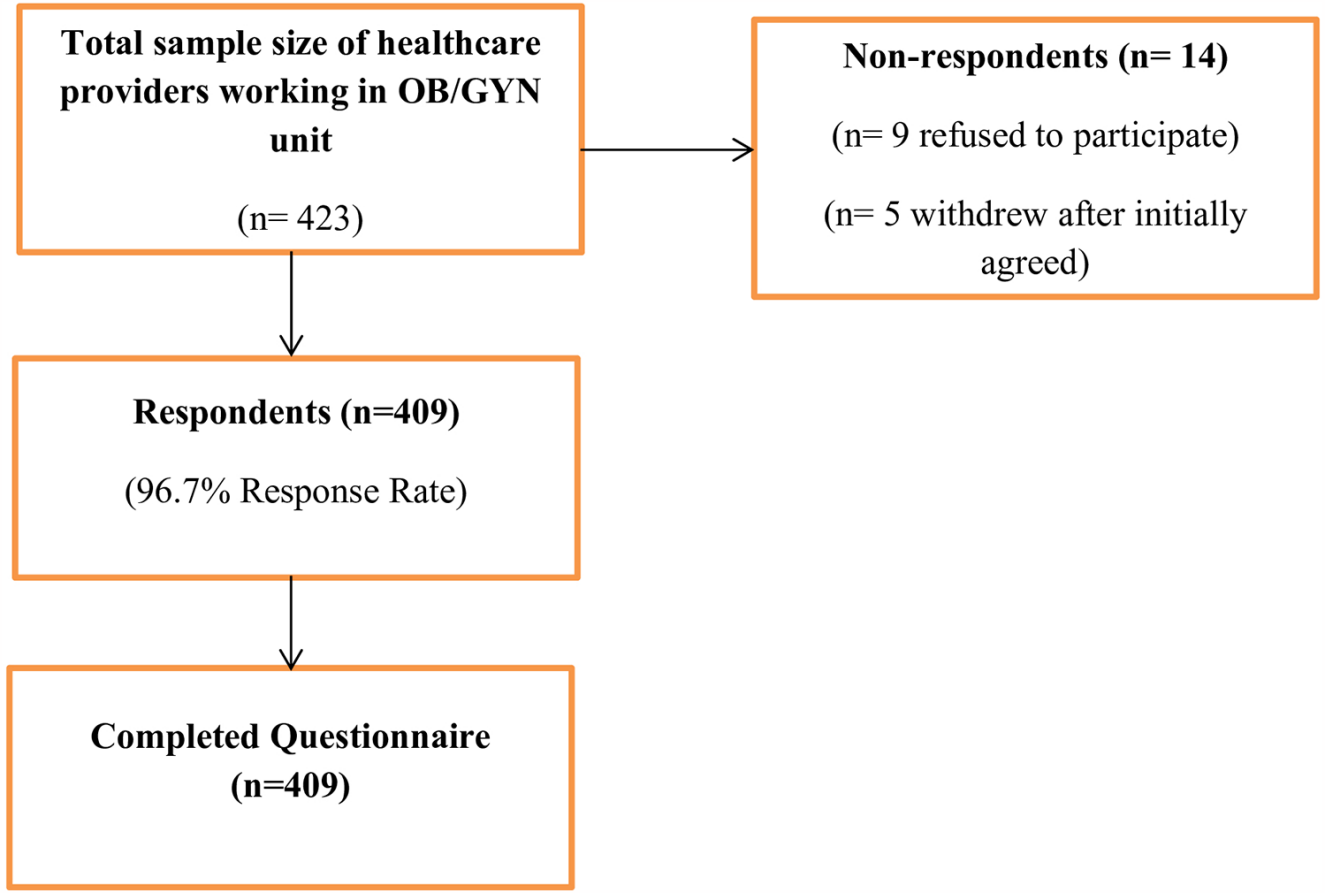
Flow diagram of participant recruitment and response.

From a total of 423 samples, 409 HCPs in the OBY/GYN unit completed the questionnaire, with a response rate of 96.7%. Out of the 14 non-respondents, 9 healthcare providers declined to participate and 5 withdrew after initially agreeing, providing only sociodemographic data without completing the main survey. The participants did not disclose the specific reasons for either their refusal or withdrawal. Efforts were made to encourage participation, such as offering further clarification of the study’s objectives and assuring confidentiality (Figure 2).

Of the respondents, 6/10 (59.9%) were male. The mean age of the respondents was 29.9 ± 3.621 years and the mean work experience was 5.74 ± 2.963 years. Regarding the respondents’ marital status and religion, 252 (61.6%) were married and 326 (79.7%) were followers of the orthodox religion. Of the participants, three-quarters (75.3%) and 328 (80.2%) were midwives and bachelor degree holders, respectively. Most of the study participants (*n* = 327, 80%) had not received training related to intimate partner violence (Table 3).

**Table 3:** Sociodemographic characteristics of healthcare providers working in OBY/GYN units in Amhara regional state referral hospitals, 2023 (*n* = 409).

Variable	Category	Frequency	Percentage
Gender	Male	245	59.9
	Female	164	40.1
	Total	409	100
Age	≤30 years	270	66
	30–40 years	133	32.5
	≥40 years	6	1.5
	Total	409	100
Religion	Orthodox	326	79.7
	Muslim	50	12.2
	Protestant	33	8.1
	Total	409	100
Marital status	Single	157	38.4
	Married	252	61.6
	Total	409	100
Profession	General practitioner	56	13.7
	Midwife	308	75.3
	Nurse	45	11
Level of education	Masters	41	10
	Bachelor	328	80.2
	Diploma	40	9.8
	Total	409	100
Work experience	≤5 years	214	52.3
	5–10 years	172	42.1
	≥11 years	23	5.6
	Total	409	100
Trained on IPV	Yes	82	20
	No	327	80.0

### Attitudes of HCPs working in OBY/GYN unit toward screening for IPV among women of reproductive age

Nearly two-thirds (63.6%) of the study participants agreed that screening for IPV could lead to the identification of patients who experienced IPV. Of the study participants, 98 (24%) strongly agreed that dealing with IPV is relevant not only to healthcare but also to the fields of law enforcement and justice.

Of the respondents, 178 (43.5%) agreed that victims of IPV would deny they were affected if they were asked about the issue (Table 4). About half (*n* = 210, 51.3%) of the study respondents had favorable attitudes toward intimate partner violence screening among the reproductive-aged women.

**Table 4:** Attitudes of HCPs working in OBY/GYN units toward screening for IPV among women of reproductive age in Amhara regional state referral hospitals, 2023 (*n* = 409).

Questions	Strongly disagree	Disagree	Neutral	Agree	Strongly agree	Total
Screening for IPV could lead to identification of patients experiencing of IPV	24 (5.9%)	48 (11.7%)	28 (6.8%)	260 (63.6%)	49 (12.0%)	409 (100%)
It is not important to screen for IPV because it is socially accepted problem	82 (20.1%)	183 (44.7%)	34 (8.3%)	100 (24.5%)	10 (2.4%)	409 (100%)
If asked, most victims of violence will deny exposure	27 (6.6%)	121 (29.6%)	63 (15.4%)	178 (43.5%)	20 (4.9%)	409 (100%)
Asking about IPV may seem offensive to most victims of violence	29 (7.1%)	128 (31.3%)	57 (13.9%)	183 (44.7%)	12 (2.9%)	409 (100%)
Screening for IPV can put abused cases in more danger	58 (14.2%)	156 (38.1%)	65 (15.9%)	119 (29.1%)	11 (2.7%)	409 (100%)
IPV is normal among couples going through marital difficulties	61 (14.9%)	197 (48.2%)	56 (13.7%)	84 (20.5%)	11 (2.7%)	409 (100%)
Healthcare professionals do not have any role except treating physical injuries caused by IPV	99 (24.2%)	229 (56.0%)	28 (6.8%)	42 (10.3%)	11 (2.7%)	409 (100%)
A women should tolerate violence to keep her family together	92 (22.5%)	161 (39.4%)	62 (15.2%)	79 (19.3%)	15 (3.7%)	409 (100%)
Dealing with violence is pertinent not only to the fields of police and justice, but also to health	21 (5.1%)	23 (5.6%)	18 (4.4%)	249 (60.9%)	98 (24.0%)	409 (100%)

### Knowledge of HCPs working in OBY/GYN unit toward screening for IPV

Most of the study participants (92.7%) noticed that IPV could occur in all settings, among all socioeconomic, religious, and cultural groups. Of the respondents, 7/10 (71.9%) reported that sexual intercourse without a woman’s permission because of fear of a partner is IPV (Table 5). Approximately 3/5 (58.4%) study participants had good knowledge of IPV.

**Table 5:** Knowledge of HCPs working in OBY/GYN units about IPV in Amhara regional state referral hospitals, 2023 (*n* = 409).

Questions	Yes	No	Total
IPV can occur in all settings, among all socioeconomic, religious and cultural groups	379 (92.7%)	30 (7.3%)	409 (100%)
Being slapped, pushed, shoved or pulled, hit, choked or burnt on purpose is IPV	330 (80.7%)	79 (19.3%)	409 (100%)
Being physically forced to have sexual intercourse when a woman did not want to is IPV	280 (68.5%)	129 (31.5%)	409 (100%)
Sexual intercourse when a woman did not want to because of fear of a partner is IPV	294 (71.9%)	115 (28.1%)	409 (100%)
Being forced to do something sexual that is degrading or humiliating is IPV	292 (71.4%)	117 (28.6%)	409 (100%)
Insulting, humiliating in front of other people, scare or intimidate her on purpose, threatened to hurt someone she cared about is IPV	331 (80.9%)	78 (19.1%)	409 (100%)
IPV might be caused by alcohol drinking	342 (84.8%)	62 (15.2%)	409 (100%)
IPV never happens during pregnancy	82 (20%)	327 (80%)	409 (100%)
IPV in pregnancy cannot cause adverse health outcome for the pregnant woman or baby	110 (26.9%)	299 (73.1%)	409 (100%)
We cannot suspect IPV unless we see physical signs/injuries and bruises	85 (20.8%)	324 (79.2%)	409 (100%)

### Healthcare providers’ responses to health setting factors in OBY/GYN unit

Less than one-third (27.9%) of the study respondents outlined the presence of intimate partner violence guidelines in their working environment. Of the study participants, 120 (29.3%) mentioned the presence of posters, brochures, or flyers to provide information about IPV, which helps maintain a sense of autonomy in how to discuss violence with a patient in their working area, and 298 (72.9%) mentioned the presence of a registration mechanism for IPV cases.

### Healthcare providers’ readiness and response to DVHCPSS domains in OBY/GYN units

Of the study participants, 190 (46.5%) (95% CI: 42–51) were ready to screen for intimate partner violence against reproductive-aged women.

More than half (51.3%) of the study participants scored below the mean value of perceived self-efficacy domain items. Of the participants, 223 (54.5%) had professional role resistance/fear of offending the patient, and nearly half (49.6%) blamed the victim for being abused (Table 6).

**Table 6:** Healthcare providers’ readiness and responses to DVHCPSS domains in OBY/GYN units of Amhara regional state referral hospitals, 2023 (*n* = 409).

Variable	Ready			Not ready		Total
Ready to screen IPV against reproductive-aged women	190 (46.5%)			219 (53.5%)		409 (100%)
Items of DVHCPSS domains	Strongly disagree	Disagree	Neutral	Agree	Strongly agree	Total
Perceived self-efficacy domain						
There are strategies I can use to encourage batterers to seek help	41 (10.0%)	118 (28.9%)	65 (15.9%)	140 (34.2%)	45 (11.0%)	409 (100%)
There are strategies I can use to help victims of IPV change their situation	31 (7.6%)	119 (29.1%)	84 (20.5%)	145 (35.5%)	30 (7.3%)	409 (100%)
I feel confident that I can make appropriate referrals for batterers	18 (4.4%)	97 (23.7%)	57 (13.9%)	191 (46.7%)	46 (11.3%)	409 (100%)
I feel confident that I can make the appropriate referrals for abused patients	14 (3.4%)	89 (21.8%)	41 (10.0%)	212 (51.8%)	53 (13.0%)	409 (100%)
I have ready access to information detailing management of IPV	62 (15.2%)	166 (40.6%)	96 (23.5%)	71 (17.4%)	14 (3.4%)	409 (100%)
There’re ways I can ask batterers about their behavior that will minimize risk to the potential victim	42 (10.3%)	161 (39.4%)	114 (27.8%)	84 (20.5%)	8 (2.0%)	409 (100%)
Professional role resistance/fear of offending the patients domain						
Asking patients about intimate partner violence is an invasion of their privacy.	114 (27.9%)	189 (46.2%)	49 (12.0%)	47 (11.5%)	10 2.4%)	409 (100%)
It is demeaning to patients to question them about abuse.	138 (33.7%)	188 (46.0%)	35 (8.6%)	46 (11.3%)	2 (0.5%)	409 (100%)
If I ask non-abused patients about IPV, they will get very angry.	53 (13.0%)	164 (40.1%)	103 (25.2%)	83 (20.3%)	6 (1.4%)	409 (100%)
I am afraid of offending the patient if I ask about IPV.	58 (14.2%)	206 (50.4%)	45 (11.0%)	93 (22.7%)	7 (1.7%)	409 (100%)
I think that investigating the underlying cause of a patient’s injury is not part of medical care.	129 (31.5%)	233 (57.0%)	15 (3.7%)	26 (6.4%)	6 (1.5%)	409 (100%)
If patients do not reveal abuse to me, then it is none of my business.	98 (24.0%)	184 (45.0%)	31 (7.6%)	84 (20.5%)	12 (2.9%)	409 (100%)
Blame victim domain						
A victim must be getting something out of the abusive relationship, or else she would leave.	107 (26.2%)	177 (43.3%)	55 (13.4%)	62 (15.1%)	8 (2.0%)	409 (100%)
I have patients whose personalities cause them to be abused.	26 (6.4%)	86 (21.0%)	62 (15.2%)	203 (49.6%)	32 (7.8%)	409 (100%)
Women who choose to step out of traditional roles are a major cause of IPV.	30 (7.3%)	79 (19.3%)	80 (19.6%)	182 (44.5%)	38 (9.3%)	409 (100%)
The victim’s passive-dependent personality often leads to abuse.	23 (5.6%)	86 (21.0%)	46 (11.3%)	203 (49.6%)	51 (12.5%)	409 (100%)
The victim has often done something to bring about violence in the relationship.	42 (10.3%)	163 (39.8%)	82 (20.1%)	104 (25.4%)	18 (4.4%)	409 (100%)
System support domain						
I have ready access to social workers or community advocates to assist in the management of IPV.	3 (0.7%)	115 (28.1%)	126 (30.8%)	140 (34.2%)	25 (6.1%)	409 (100%)
I feel that social work personnel can help manage IPV patients.	1 (0.2%)	50 (12.2%)	43 (10.5%)	236 (57.7%)	79 (19.3%)	409 (100%)
I have ready access to mental health services should our patients need referrals.	1 (0.2%)	110 (26.9%)	102 (24.9%)	168 (41.1%)	28 (6.9%)	409 (100%)
I feel that the mental health services at my clinic or agency can meet the needs to IPV victims.	2 (0.4%)	71 (17.4%)	48 (11.7%)	206 (50.4%)	82 (20.1%)	409 (100%)
Victim provider safety domain						
There is no way to ask batterers about their behaviors without putting the victims in more danger.	62 (15.1%)	204 (49.9%)	45 (11.0%)	87 (21.3%)	11 (2.7%)	409 (100%)
I am afraid if I talk to the batterer, I will increase risk for the victim.	35 (8.6%)	165 (40.3%)	46 (11.3%)	149 (36.4%)	14 (3.4%)	409 (100%)
I feel it is best to avoid dealing with the batterer out of fear and concern for the victim’s safety.	60 (14.7%)	148 (36.2%)	39 (9.5%)	154 (37.7%)	8 (1.9%)	409 (100%)
I am reluctant to ask batterers about their abusive behavior out of concern for my personal safety.	61 (14.9%)	163 (39.9%)	80 (19.6%)	99 (24.2%)	6 (1.4%)	409 (100%)
There is not enough security at my work place to safely permit discussion of IPV with batterers.	32 (7.8%)	104 (25.4%)	61 (14.9%)	190 (46.5%)	22 (5.4%)	409 (100%)
I am afraid of offending patients if I ask about their abusive behavior.	43 (10.5%)	174 (42.5%)	52 (12.7%)	126 (30.8%)	14 (3.4%)	409 (100%)
When challenged, batterers frequently direct their anger toward healthcare providers.	22 (5.4%)	111 (27.1%)	96 (23.5%)	158 (38.6%)	22 (5.4%)	409 (100%)

### Factors affecting HCPs’ readiness to screen for IPV among reproductive-aged women in OBY/GYN units

On crude bivariable analysis, the factors found to be significantly associated with the readiness of healthcare providers’ in the OBY/GYN unit were gender, age, marital status, profession, training on IPV, attitude toward IPV screening, knowledge of IPV, and availability of IPV guidelines in the working environment.

From the eight variables eligible for the multivariable analysis, gender, training on IPV, attitudes toward IPV screening, knowledge of IPV, and availability of IPV guidelines in the working environment were found to be significantly associated (Table 7).

**Table 7:** Bivariable and multivariable analysis of factors affecting healthcare providers’ readiness to screen for intimate partner violence among reproductive-aged women in obstetrics and gynecology units of Amhara regional state referral hospitals, 2023 (*n* = 409).

Variable	Categories	Readiness to screen IPV		COR (95% CI)	AOR (95% CI)
		Yes	No		
Gender	Male	125	120	1.59 (1.06–2.37)*	**1.64 (1.03–2.61)** *
	Female	65	99		**1**
Age	≤30 years	117	153	0.382 (0.069–2.12)	0.24 (0.04–1.46)
	30–40 years	69	64	0.539 (0.095–3.04)	0.33 (0.05–2.05)
	≥40 years	4	2		**1**
Marital status	Single	79	78	1.29 (0.86–1.92)	1.30 (0.80–2.08)
	Married	111	141		**1**
Profession	General practitioner	35	21	3.02 (1.34–6.83)**	1.67 (0.67–4.18)
	Midwife	139	169	1.49 (0.78–2.86)	1.47 (0.71–3.05)
	Nurse	16	29		**1**
Have you taken training on IPV	Yes	56	26	3.10 (1.85–5.19)***	**2.84** (1.64–4.94)***
	No	134	193		**1**
Attitude	Favorable	125	85	3.03 (2.02–4.54)***	**2.21** (1.42–3.44)***
	Unfavorable	65	134		**1**
Knowledge	Good	136	103	2.84 (1.88–4.28)***	**2.23** (1.42–3.50)**
	Poor	54	116		**1**
Availability of IPV guideline	Yes	70	46	2.19 (1.42–3.40)***	**1.74** (1.07–2.81)*
	No	120	173		**1**

1, Reference category; COR, crude odds ratio; AOR, adjusted odds ratio; CI, confidence interval.

* *p* < 0.05; **p < 0.01; ***p < 0.001.

The odds of readiness to screen for IPV were 1.6 times higher among male participants compared with their female counterparts [adjusted odds ratio (AOR) = 1.64, 95% CI: 1.03–2.61].

Participants who had been trained in IPV were 2.8 times more likely to be ready to screen for IPV compared to those who had no training in IPV (AOR = 2.84, 95% CI: 1.64–4.94). Healthcare providers with favorable attitudes toward IPV screening were 2.2 times more likely to be ready to screen for IPV against reproductive-aged women compared with those participants with unfavorable attitudes (AOR = 2.21, 95% CI: 1.42–3.44).

The study respondents with good knowledge of IPV were 2.2 times more likely to report readiness to screen IPV compared to participants with poor knowledge of IPV (AOR = 2.23, 95% CI: 1.42–3.50).

Availability of IPV guidelines in the working environment was also another positively associated variable with readiness to screen IPV. Those healthcare providers who had an IPV guideline in their working environment were 1.7 times more ready to screen IPV compared to those who did not have guidelines (AOR = 1.74, 95% CI: 1.07–2.81).

## Discussion

This study assessed the readiness of healthcare providers’ and associated factors to screen for intimate partner violence in OBY/GYN units against reproductive-aged women in Amhara regional state referral hospitals, northwest Ethiopia.

The findings revealed that the proportion of healthcare providers’ readiness to screen for IPV in the OBY/GYN unit was found to be 46.5% (95% CI: 42–51). Factors associated with readiness to screen for IPV included being male, having training experience in IPV, having favorable attitudes toward IPV screening, having knowledge of IPV, and availability of IPV guidelines in the work environment.

According to this study, less than half of healthcare providers were ready to screen for IPV. This might be due to more than half of the study participants having poorly perceived self-efficacy in handling IPV, over half of the study participants having poor system support, and more than half of the study participants having professional role resistance/fear of offending the patient. This explanation is supported by a study carried out in Jeddah, Saudi Arabia, which found that having professional resistance/fear of offending the patient and a lack of psychiatric support reduced dentists’ readiness to screen for IPV (42). A study carried out in Sweden stated that HCPs who screened for IPV had higher self-efficacy, higher availability of support networks for IPV, and lower conflicting professional roles and fears regarding IPV screening (43). Therefore, to have enough ready HCPs and to avoid missing opportunities to screen and support women who experience violence, it is necessary to improve their self-efficacy and system support and reduce their fear of offending the patient. Having self-efficacy in one’s ability may influence the course of action one takes in a given situation.

As screening for IPV and giving care to its victims are expected to be highly correlated, preparedness can be affected by almost similar variables. In addition, factors affecting preparedness to provide IPV care in a study conducted in Tanzania were found to affect readiness to screen for IPV in this study. Considering this, when we compare the proportion of healthcare providers’ readiness to screen for IPV, it was found to be lower compared to the findings of a study from Tanzania (54%) (44). This disparity might be due to the different tools used to measure the outcome variable. Similarly, the readiness of HCPs to screen for IPV in this study was lower compared to that in a study from Uganda, where 56% of healthcare providers screened pregnant women for IPV (45). Readiness to screen for IPV directly impacts the likelihood of actual screening practice. These disparities might be due to political instability. HCPs in this study area might be impacted by the societal and economic challenges left by the conflict between the Ethiopian federal government and the Tigrai People’s Liberation Front (TPLF), such as increased workload, resource constraints, and community trauma, which may have influenced their readiness to address sensitive issues like IPV (46, 47). Therefore, it is essential to design and implement strategies that mitigate the effects of political instability on healthcare systems. Governments and stakeholders must also invest in creating stable political environments by enhancing peace-building initiatives, addressing the root causes of conflicts, and ensuring equitable resource distribution to prevent future instability. Collaborative efforts between the government, non-governmental organizations, and international partners are critical to achieving long-term stability and improving the resilience of healthcare systems in conflict-affected areas, since the prevalence of IPV is increased during periods of political instability (48, 49).

The results of the present study identified gender as one of the positively associated variables with healthcare providers’ readiness to screen for IPV. Male healthcare providers had higher odds of readiness to screen for IPV compared with their female counterparts (AOR = 1.64, 95% CI: 1.03–2.61). These findings were supported by a study conducted in East Gojjam, Ethiopia, in which male nurses were more likely to give care for women exposed to IPV (30). This finding contradicts results from a study conducted among dental healthcare providers in Jeddah, Saudi Arabia, which showed lower odds of readiness to screen for IPV among male healthcare providers (42). This contradiction may be due to cultural differences in the acceptability of men asking women about personal matters, as well as variations in the gender distribution of participants. In the Jeddah study, 54% of participants were female, whereas in our study, 60% were male. In addition, differences in outcome measurements may also contribute to this difference. Therefore, it is crucial to consider cultural norms and gender dynamics when designing strategies and intervention programs. It is essential to create gender-sensitive approaches to foster trust and comfort in patient–provider interactions.

In this study, participants who trained in IPV were 2.8 times more likely to be ready to screen for IPV (AOR = 2.84, 95% CI: 1.64–4.94). This finding is supported by studies carried out in Tanzania (44) and Uganda (45, 50). This could be justified as educational training has been shown to effectively enhance clinicians’ knowledge and preparedness in managing IPV, and it can also improve their ability to address barriers to IPV screening (51, 52). In addition, such training can equip healthcare providers with the skills and confidence needed to effectively support patients, collaborate with colleagues, and navigate the healthcare system to combat violence and abuse (1). Therefore, it is necessary to enhance HCPs capacity for IPV screening and management. Ministries of Health and healthcare organizations should prioritize IPV screening in their policies and allocate adequate resources for its effective implementation. This includes designing and delivering consistent training programs on IPV screening and management, along with addressing each healthcare provider. Such training should be integrated into the professional development of all HCPs. IPV screening should also be routinely practiced in clinical settings, with healthcare providers equipped with the necessary tools and support to ensure effective care for IPV victims.

Healthcare providers’ who had favorable attitudes toward IPV screening had higher odds of readiness to screen for IPV compared with their counterparts with unfavorable attitudes (AOR = 2.21, 95% CI: 1.42–3.44), a finding consistent with a study conducted in Iran (53). This may be because a positive perception of IPV screening encourages healthcare professionals to engage proactively and address IPV victims. Attitudes play a significant role in shaping behavior; individuals are more likely to act when they view the action positively and believe that significant others expect them to do so (54). Therefore, attitudes toward IPV screening need to be improved to have HCPs who are prepared to do so.

This study revealed that participants with a good knowledge of IPV had higher odds of readiness to screen for IPV compared to participants with a poor knowledge of IPV (AOR = 2.23, 95% CI: 1.42–3.50). This aligns with findings from East Gojjam zone in Ethiopia, where nurses lacking IPV knowledge were less likely to provide care to women experiencing IPV (30). Healthcare providers with good knowledge of IPV are more likely to understand the effects of IPV and recognize the potential benefits of IPV screening. Their knowledge may also help contribute to a better perspective on prevention efforts. The results show a clear connection between knowledge and its practical application (55). Therefore, improving HCPs’ knowledge about IPV, its effects, and prevention through different techniques, such as preparing campaigns and consistent training, plays a crucial role in their readiness to screen for IPV.

The availability of IPV guidelines in the work environment was also significantly associated with increased readiness among HCPs to screen for IPV (AOR = 1.74, 95% CI: 1.07–2.81). This finding is supported by a study conducted in Tanzania (44). The presence of standardized protocols is important to guide service delivery (3). Evidence also suggests that the lack of such guidelines and protocols is a contributing factor to high maternal and neonatal mortality rates in resource-limited settings (56). Therefore, policymakers should prioritize the development, availability, and implementation of standardized IPV screening protocols in healthcare environments. National health policies should integrate IPV screening into routine care, allocate sufficient resources for HCP training, and support continuous professional development. In addition, healthcare providers should consistently adhere to IPV screening guidelines.

### Limitations of the study

Although conducting a pilot study to assess the reliability and construct validity of the DVHCPSS tool was a strength of the study, the sample size for the pilot study was limited due to resource constraints. A larger sample size might have allowed for the inclusion of additional variables that were excluded during the validation process. In addition, this study was conducted exclusively in tertiary hospitals, which are typically better equipped and staffed with higher-level HCPs. As a result, the findings may not be fully generalizable to other healthcare settings within the region, such as primary and secondary healthcare facilities. Future research could address this limitation by including healthcare providers from all levels of care, which would offer more comprehensive and generalizable results.

## Conclusion

In this study, fewer than half of healthcare providers in the OBY/GYN unit were ready to screen for IPV. Most healthcare providers reported poor perceived self-efficacy and professional role resistance/fear of offending the patient, with nearly half blaming the victim for the abuse. Factors contributing to readiness to screen for IPV included a favorable attitude toward IPV screening, good knowledge of IPV, the gender of the HCP, training on intimate partner violence, and the availability of IPV guidelines in the workplace. Based on these findings, it is recommended that stakeholders provide consistent, comprehensive, and updated training for HCPs at all levels of care. In addition, standardized guidelines and protocols, including safety measures and validated screening tools, should be made available. Healthcare facilities must also be adequately equipped to support meticulous screening practices. Integrating IPV screening, management, and rehabilitation services with other reproductive health services can ensure a holistic approach to IPV screening and care. Finally, implementing monitoring and evaluation mechanisms will help assess the quality and effectiveness of IPV screening services, fostering continuous improvement. These measures can enhance the readiness of HCPs and improve care for IPV survivors.

## Data Availability

The raw data supporting the conclusions of this article will be made available by the authors, without undue reservation, to any qualified researcher upon reasonable request.
